# ChatGPT as a Source for Patient Information on Patellofemoral Surgery—A Comparative Study Amongst Laymen, Doctors, and Experts

**DOI:** 10.3390/clinpract14060186

**Published:** 2024-11-05

**Authors:** Andreas Frodl, Andreas Fuchs, Tayfun Yilmaz, Kaywan Izadpanah, Hagen Schmal, Markus Siegel

**Affiliations:** 1 Department of Orthopedic Surgery and Traumatology, Freiburg University Hospital, Albert Ludwigs University Freiburg, Hugstetter Straße 55, 79106 Freiburg, Germany; 2 Department of Orthopedic Surgery, University Hospital Odense, Sdr. Boulevard 29, 5000 Odense, Denmark

**Keywords:** patellofemoral joint, ChatGPT, healthcare AI

## Abstract

Introduction: In November 2022, OpenAI launched ChatGPT for public use through a free online platform. ChatGPT is an artificial intelligence (AI) chatbot trained on a broad dataset encompassing a wide range of topics, including medical literature. The usability in the medical field and the quality of AI-generated responses are widely discussed and are the subject of current investigations. Patellofemoral pain is one of the most common conditions among young adults, often prompting patients to seek advice. This study examines the quality of ChatGPT as a source of information regarding patellofemoral conditions and surgery, hypothesizing that there will be differences in the evaluation of responses generated by ChatGPT between populations with different levels of expertise in patellofemoral disorders. Methods: A comparison was conducted between laymen, doctors (non-orthopedic), and experts in patellofemoral disorders based on a list of 12 questions. These questions were divided into descriptive and recommendatory categories, with each category further split into basic and advanced content. Questions were used to prompt ChatGPT in April 2024 using the ChatGPT 4.0 engine, and answers were evaluated using a custom tool inspired by the Ensuring Quality Information for Patients (EQIP) instrument. Evaluations were performed independently by laymen, non-orthopedic doctors, and experts, with the results statistically analyzed using a Mann–Whitney U Test. A *p*-value of less than 0.05 was considered statistically significant. Results: The study included data from seventeen participants: four experts in patellofemoral disorders, seven non-orthopedic doctors, and six laymen. Experts rated the answers lower on average compared to non-experts. Significant differences were observed in the ratings of descriptive answers with increasing complexity. The average score for experts was 29.3 ± 5.8, whereas non-experts averaged 35.3 ± 5.7. For recommendatory answers, experts also gave lower ratings, particularly for more complex questions. Conclusion: ChatGPT provides good quality answers to questions concerning patellofemoral disorders, although questions with higher complexity were rated lower by patellofemoral experts compared to non-experts. This study emphasizes the potential of ChatGPT as a complementary tool for patient information on patellofemoral disorders, although the quality of the answers fluctuates with the complexity of the questions, which might not be recognized by non-experts. The lack of personalized recommendations and the problem of “AI hallucinations” remain a challenge. Human expertise and judgement, especially from trained healthcare experts, remain irreplaceable.

## 1. Introduction

In November 2022 OpenAI launched ChatGPT for public use through a free online platform [1]. ChatGPT is an artificial intelligence (AI) chatbot trained on a broad dataset containing a wide range of different topics, including medical literature [1,2]. The chatbot can provide well-formulated and seemingly well-informed answers, when prompted with inquiries. The usability and quality of AI-generated responses are discussed widely and are the subject of current investigations [3,4].

Pain caused by the patellofemoral joint is one of the most common conditions amongst young adults and patients often seek advice for their condition [5]. The underlying reasons for referred patellofemoral pain are numerous [6]. It is mostly caused by abnormal mechanical joint loading during exercise and daily activities, for example, due to patellofemoral instability [7]. Thus, secondary pathologies such as chondral defects can arise, prompting further, even surgical treatment.

Patients often seek sources of information before and after dialogue with their treating doctor, especially if surgery is mentioned in case of a special medical condition. Thus, amongst the various sources found through social media and the internet itself, the former “doctor google” may be replaced by ChatGPT, as the number of users grows rapidly.

ChatGPT was trained using language patterns with specific subroutines to generate responses that are as human-like as possible. These language models, such as in the case of ChatGPT-3, were trained using 75 billion words and approximately 175 billion different parameters from online data [8]. This makes it possible to cover a wide range of fields and topics, such as healthcare and medicine. The quality of some responses, especially regarding medical questions, has recently been intensively studied [2,4,9,10,11]. When it comes to surgical activities, it is essential to create a detailed picture based on individual patient data (such as pre-existing conditions, imaging findings, previous surgeries, etc.) and to determine further therapeutic and, if necessary, surgical procedures based on this information. Given the large amounts of data, the focus is on evaluating this data as quickly as possible while maintaining reliability and competence. ChatGPT seems to offer an option for data processing. Not only can large datasets be quickly analyzed, but the AI is also capable of recognizing patterns and providing easily understandable, personalized responses within seconds. The chatbot learns interactively and continuously from each user, leading to increasingly personalized answers [8,12].

In this study we examine the quality of ChatGPT as a source of information regarding patellofemoral conditions and surgery. We hypothesize that there will be differences in the evaluation of the responses generated by ChatGPT between populations with different levels of expertise in patellofemoral disorders.

## 2. Methods

From April to July 2024 we conducted a comparison between laymen (non-medical professionals), doctors (non-orthopedics), and experts in patellofemoral disorders on the basis of a list of 12 questions. Non-orthopedic doctors included those with specialties in gynecology, general surgery, anesthesiology, internal medicine, and urology. Each of them were chosen randomly. The questions used, included six questions aiming for the description of a femoropatellar topic and six aiming for a recommendation for the treatment of patellofemoral disorders, all concerning patellar instability and retropatellar osteoarthritis. The questions could each be divided into “basic” or “advanced” content. “Basic” included simple questions in terms of content, and “advanced” included questions with specific inquiries in the field. The creation of the questions and their grouping into “basic” and “advanced” was done in collaboration with experts in the field of patellofemoral disorders. These experts also ultimately formed the “experts” participant group. The questions used to prompt ChatGPT are listed in Table 1.

Questions were typed into ChatGPT in April 2024 using the ChatGPT 4.0 engine, and the answer to each question was noted. Similar to the Ensuring Quality Information for Patients (EQIP) tool, we constructed an evaluation tool. The answers given by ChatGPT were evaluated in four fields: compatibility/coherence, thoroughness, style [13,14], and overall quality. For each field of evaluation, between 1–10 points could be given, where 10 points symbolized the highest satisfaction for each aspect. Thus, a maximum score of 40 points or minimum of 4 points could be reached (Table 2).

The questions shown in Table 1 were presented to ChatGPT by the authors. The resulting answers were recorded in a separate document, containing the questions and their corresponding answers. This document formed the basis for the evaluations carried out by the groups.

Evaluation of the same question–answer constellation was done independently by laymen, non-orthopedic doctors, and experts in patellofemoral surgery. The results were then compared and statistically analyzed with a Mann–Whitney U Test. A *p*-value of less than 0.05 was considered statistically significant. Statistical analyses were performed using IBM SPSS Statistics version 29.0.0.0 (IBM Corp., Armonk, New York, NY, USA). The results of all the statistical tests were interpreted in an exploratory sense.

## 3. Results

Overall, we included the data of seventeen participants. Of those, four were experts in patellofemoral surgery, seven non-orthopedic doctors, and six laymen. The point values of the individual evaluation criteria were summarized for each answer and group, and an average score per answer was calculated for each evaluator group. The breakdown of the values can be found in Table 3.

Among the evaluator groups, the experts gave the lowest average score per answer, whereas the doctors and laymen nearly equaled in their average scores. An intraclass correlation coefficient (ICC) was calculated, which showed a very good accordance of rater evaluation in the expert group and amongst the laymen and non-orthopedic doctors [15]. We calculated an ICC of 0.97 (*p* = 0.001) for the experts and an ICC of 0.99 (*p* = 0.001) for the non-expert group. Thus, for the later sub-analysis of the descriptive and recommendatory answers, the groups “doctors” and “laymen” were analyzed as one group. The results were then compared to the average points per answer of the expert group. The mean average of points was 29.3 ± 5.8 for the experts and 35.3 ± 5.7 for the non-experts (Table 3).

Table 4 shows the analysis of the descriptive answers and the sub-analysis of questions with “basic” and “advanced” complexity. A significantly lower average of points was seen in the expert group. The overall mean for all descriptive answers was 28.2 ± 6.3 in the expert group, whereas a mean of 35.3 ± 6.0 was noted for the non-expert group. As the complexity of the questions increased, the answers from ChatGPT were rated lower on average by the expert group. The answers to the questions classified as “basic” received an average score of 29.7 ± 5.1 points, while the answers to the “advanced” questions received an average score of 26.7 ± 7.2. This trend was not observed in the non-expert group, where the average scores for both classes of answers showed no statistical differences (“basic”: 35.3 ± 5.7; “advanced”: 35.4 ± 6.3).

The evaluation of all answers with a recommendatory character is shown in Table 5. The experts scored the answers, on average, with fewer points than the non-expert group. This was documented significantly, with the exception of two answers (A7 *p* = 0.87 and A11 *p* = 0.30). The overall mean of the points was 30.5 ± 5.1 points from the experts and 35.3 ± 5.4 points from the non-expert group. Similar to the analysis of the descriptive answers, with the increasing complexity of the questions, the answers were rated lower on average by the experts (“basic”: 31.9 ± 4.3; “advanced”: 29.0 ± 5.6). In the non-expert group, the average scores for both classes of answers were 35.3 ± 5.9 points for “basic” classified and 35.2 ± 4.9 points for ”advanced” classified questions.

## 4. Discussion

The main findings of the present study are that ChatGPT provides good quality answers to questions in the context of patellofemoral disorders; however, the more complex the questions, the lower the ratings given by the expert group, whereas this was not observed for the non-orthopedic doctors and laymen (non-experts). ChatGPT is a good tool as a source of information on disorders of the patellofemoral joint, but the quality of the answers depends on the complexity and the quality (descriptive/recommendatory) of the question, and this does not appear to be recognized by people from outside the field.

The integration of AI into the medical field, especially for patient education and as a source of information for patients, seems to offer promising advantages. However, in the last two years the utilization of AI, especially ChatGPT, in healthcare has been investigated, particularly for internal medicine and surgical disciplines [3,16,17,18]. Studies exist suggesting ChatGPT as a source for patient education on total hip arthroplasty, sports medicine, and pediatric orthopedics [19,20,21]. However, its lack of reliability and inability to provide personalized recommendations regarding orthopedic treatments have been identified as problematic [22,23]. With the tasks/questions with higher complexity that were used to prompt ChatGPT, these observations have been made in this study as well, especially if the character of the question typed in was recommendatory.

In this context, the questionable reliability of ChatGPT’s responses and the sources it uses to generate these responses are frequently highlighted [1,4,24]. This results in two fundamental issues. Firstly, ChatGPT’s large language models (LLMs) were trained on data up to September 2021. Secondly, the algorithm arbitrarily relies on publicly accessible resources for generating responses, instead of using professionally validated sources [25]. Thus, the answers given by early versions of ChatGPT lack the developments in knowledge and research that occurred after September 2021, and the lack of citations where information has been gathered might lead to a biased representation and influence on the patient [26].

ChatGPT has the potential to be a valuable tool for patient information in preoperative and postoperative phases. However, the results of this study show its capability to obtain surface-level information on various patellofemoral topics. It can give simple explanations and can explain complex medical terms in a fast, concise, and simple way, offering a better insight to patients regarding their medical conditions [8,27]. Although ChatGPT is designed to provide helpful suggestions, it misses regulatory mechanisms to control the correctness of its answers. Thus, an incorrect response may be displayed as potentially correct by the LLM, a condition also referred to as “AI hallucination” [28,29]. As LLMs are a series of mathematical implementations on the basis of a statistical pattern rather than a conscious process, the model may emphasize certain parts of the input, while neglecting potentially more relevant parts [30]. With the update in 2023 and changes to the 4.0 engine, some of the issues named above have been addressed. The new engine features a more advanced architecture, reportedly utilizing over a trillion parameters. This increase in parameters allows GPT-4 to handle more complex tasks and generate more accurate responses. Thus, ChatGPT 4.0. is less prone to “hallucinations” (producing incorrect or nonsensical information) and it generally provides more factual and unbiased responses [31].

In a comparative analysis between the existing versions, particularly versions 3.0 and 4.0, Srinivasan et al. examined the response quality in the field of bariatric surgery. They presented each version of ChatGPT with FAQs about bariatric procedures and compared the responses. The analysis revealed the clear superiority of ChatGPT 4.0 compared to its predecessors [32]. Similar results were reported by the Japanese research group led by Nakajima et al. They tested the “knowledge” of ChatGPT versions 3.5, 4.0, and 4 V by having the AIs take the Japanese Board of Orthopaedic Surgery Examination. Among the tested versions, only ChatGPT 4.0 reached the passing threshold [33]. In addition to ChatGPT, there are other AIs such as Gemini, BARD, and CoPilot, whose usability in the medical sector has also been studied, sometimes even comparatively. Hanci et al. compared the aforementioned AIs in the field of palliative care. In their analysis, particular emphasis was placed on the comprehensibility and quality of the responses [34]. The authors evaluated the quality of the responses based on the JAMA (Journal of the American Medical Association) benchmark and the DISCERN criteria. Among the five chatbots compared, all generally provided satisfactory responses, although the final language level of the responses was above the desired simple language level.

In the field of orthopedics, comparative studies have also been conducted on the applicability of AIs. Fabijan et al. had ChatGPT 4.0, Gemini, CoPilot, PopAI, and YouChat determine the Cobb angle for monoconvex scoliosis and provide corresponding treatment recommendations [35]. Among the tested AIs, the determinations and recommendations from ChatGPT, CoPilot, and PopAI were found to be accurate, whereas Gemini and YouChat produced weaker results. However, both studies—by Hanci et al. and Fabijan et al.—ultimately emphasized that the application of AI in medicine can be seen as a major advantage in the future, but in its current versions, it still requires reliable human oversight and review of responses.

In summary, ChatGPT, particularly version 4.0, can be effectively used for information retrieval regarding disorders of the patellofemoral joint, especially when it comes to describing factual situations. However, recommendations related to therapeutic modalities proposed by ChatGPT should be critically evaluated. Even though version 4.0 is less prone to “AI hallucinations”, this issue is not fully addressed and cannot be completely ruled out. To further reduce their susceptibility to AI hallucinations and make their response quality more reliable, AIs must be continuously trained with clinical studies to ensure that information is always presented based on the most up-to-date scientific knowledge [36]. Additionally, it is essential to implement a self-checking algorithm that independently and reliably reviews the chatbots’ responses for errors and quality deficiencies and corrects them. Once these issues are resolved, the use of AI in clinical practice can offer significant benefits for both doctors and patients. This could include creating medical reports, providing self-information before and after medical visits, or monitoring healing progress in terms of post-treatment protocols for certain injuries.

Ultimately, and most importantly, human expertise and judgement, especially that of trained medical professionals, cannot be replaced by ChatGPT to date.

## 5. Limitations

The limitations of this study clearly lie in the size of the evaluator cohort and its non-comparative style. Consequently, the statistical interpretation of the results is purely exploratory and the significance of the study is diminished as a result. Additionally, the evaluation of the responses proved challenging due to the lack of standardized tools. Therefore, a custom evaluation tool, inspired by the EQIP, had to be created. Furthermore, there are no reference values for ChatGPT’s responses. Thus, the assessments and judgments of the experts were considered as the benchmark for an optimal answer, with which the risk of confirmation bias rises. Furthermore, it must be emphasized that the results of this work pertain solely to the use of ChatGPT 4.0, and no comparisons were made with other versions or other chatbot variants.

## 6. Conclusions

ChatGPT provides good quality answers to questions concerning patellofemoral disorders, although questions with higher complexity were rated lower by patellofemoral experts compared to non-experts. This study emphasizes the potential of ChatGPT as a complementary tool for patient information on patellofemoral disorders, although the quality of the answers fluctuates with the complexity of the questions, which might not be recognized by non-experts. The lack of personalized recommendations and the problem of “AI hallucinations” remain a challenge. Human expertise and judgement, especially from trained healthcare experts, remain irreplaceable. Nevertheless, AI remains an intriguing subject and has already demonstrated its usefulness in certain aspects. With the further development of subroutines and through deep learning, future versions are likely to achieve advancements that should be analyzed for their applicability, particularly in the medical field, through comparative studies.

## Figures and Tables

**Table 1:** List of questions used to prompt ChatGPT.

**Descriptive questions with moderate complexity:**
- What is patellofemoral instability?
- What are the factors which contribute to patellofemoral maltracking?
- How can retropatellar cartilage damage resulting from patellofemoral instability be treated?
**Highly complex/Advanced:**
- What are the advantages of dynamic MPFL (Medial Patellofemoral Ligament) reconstruction over ‘traditional’ MPFL reconstruction using hamstring grafts?
- A trochlear osteotomy has been recommended to me. What are the risks of this procedure?
- I’ve been advised to undergo quadriceps training to stabilize my patella. Is this an effective treatment?
**Recommendatory questions with moderate complexity:**
- My patella was once dislocated, what should I do?
- Is MPFL reconstruction a suitable therapy for single patellar dislocation?
- Can you recommend a post-operative protocol following MPFL reconstruction using a gracilis graft?
**Highly complex/Advanced:**
- I’ve previously had a trochleoplasty with MPFL reconstruction and tuberosity osteotomy, and now I am experiencing pain in the anterior knee joint again. Which therapy is suitable for me now?
- I have a TT-TG (Tibial Tuberosity-Trochlear Groove) distance of 23 mm, trochlear dysplasia type Dejour D, and habitual patellar dislocation. Which therapy is suitable for me?
- Which is the most suitable infiltration therapy for my retropatellar arthritis?

**Table 2:** Evaluation tool according to EQIP.

Field of Evaluation	Not Applicable	Partially True (50%)	Fully True	Points
Compatibility/Coherence (Does the answer fit the question? Is the answer adequate?)	1 2 3 4	5 6 7	8 9 10	
Thoroughness (Is the answer detailed, and does it fully and [subjectively] correctly address the question?)	1 2 3 4	5 6 7	8 9 10	
Style (Are the language, structure, and style easily understandable?)	1 2 3 4	5 6 7	8 9 10	
Overall Quality (Summary assessment of the answer)	1 2 3 4	5 6 7	8 9 10	
Total score				

**Table 3:** Overview of average points per group.

		Descriptive						Recommendatory					
Reviewer Group	*n*	Mean Points A1	Mean Points A2	Mean Points A3	Mean Points A4	Mean Points A5	Mean Points A6	Mean Points A7	Mean Points A8	Mean Points A9	Mean Points A10	Mean Points A11	Mean Points A12
Experts	4	29.0 ± 5.0	27.3 ± 6.7	32.8 ± 1.7	23.3 ± 6.2	28.0 ± 9.6	28.8 ± 6.1	34.0 ± 4.9	29.5 ± 4.8	27.7 ± 2.9	30.8 ± 3.2	29.5 ± 7.9	26.8 ± 5.7
Doctors	6	34.7 ± 2.7	34.0 ± 2.4	37.2 ± 2.3	33.0 ± 7.1	34.9 ± 2.8	36.8 ± 3.5	31.5 ± 4.5	35.7 ± 2.9	38.0 ± 1.3	35.0 ± 3.0	34.0 ± 7.5	36.2 ± 3.1
Laymen	7	33.4 ± 8.6	34.7 ± 8.4	35.4 ± 6.1	34.1 ±11.1	31.5 ± 5.2	34.4 ± 4.8	31.9 ± 4.8	37.3 ± 5.6	33.6 ± 8.8	37.9 ± 3.1	33.6 ± 6.0	34.4 ± 5.1
Total	17	32.8 ± 6.3	33.6 ±7.1	35.4 ± 4.4	31.2 ± 9.6	34.9 ± 6.8	33.9 ±5.4	33.9 ± 6.1	34.9 ± 5.4	34.8 ± 6.1	35.2 ± 4.0	32.8 ± 6.8	33.2 ± 5.8

**Table 4:** Sub-analysis of descriptive answer evaluation.

		Experts (n = 4)	Non-Experts (n = 13)	*p*	*r*
basic	Mean points A1	29.0 ± 5.0	34.0 ± 6.4	0.04	0.49
	Mean points A2	27.3 ± 6.7	35.5 ± 6.2	0.03	0.51
	Mean points A3	32.8 ± 1.7	36.2 ± 4.6	0.02	0.57
advanced	Mean points A4	23.3 ± 6.2	33.6 ± 9.3	0.02	0.55
	Mean points A5	28.0 ± 9.6	37.0 ± 4.2	0.03	0.52
	Mean points A3	28.8 ± 6.1	35.5 ± 4.2	0.03	0.53

*r*: mean effect strength *r* < 0.1 weak, 0.1 < *r* < 0.5 medium, *r* > 0.5 strong.

**Table 5:** Sub-analysis of recommendatory answer evaluation.

		Experts (n = 4)	Non-Experts (n = 13)	*p*	*r*
basic	Mean points A7	34.0 ± 4.9	33.9 ± 6.6	0.87	0.04
	Mean points A8	29.5 ± 4.8	36.5 ± 4.5	0.03	0.54
	Mean points A9	27.7 ± 2.9	35.6 ± 6.7	0.06	0.47
advanced	Mean points A10	30.8 ± 3.2	33.6 ± 9.3	0.01	0.60
	Mean points A11	29.5 ± 7.9	36.5 ± 3.3	0.30	0.28
	Mean points A12	26.8 ± 5.7	33.7 ± 6.6	0.02	0.58

*r*: mean effect strength *r* < 0.1 weak, 0.1 < *r* < 0.5 medium, *r* > 0.5 strong.

## Data Availability

All data are within the manuscript, further inquiries can be directed to the corresponding author.
